# Tapped out or barely tapped? Recommendations for how to harness the vast and largely unused potential of the Mechanical Turk participant pool

**DOI:** 10.1371/journal.pone.0226394

**Published:** 2019-12-16

**Authors:** Jonathan Robinson, Cheskie Rosenzweig, Aaron J. Moss, Leib Litman

**Affiliations:** 1 Department of Computer Science, Lander College, Flushing, New York, United States of America; 2 Prime Research Solutions, Queens, New York, United States of America; 3 Department of Clinical Psychology, Columbia University, New York, New York, United States of America; 4 Department of Psychology, Lander College, Flushing, New York, United States of America; Aalborg University, DENMARK

## Abstract

Mechanical Turk (MTurk) is a common source of research participants within the academic community. Despite MTurk’s utility and benefits over traditional subject pools some researchers have questioned whether it is sustainable. Specifically, some have asked whether MTurk workers are too familiar with manipulations and measures common in the social sciences, the result of many researchers relying on the same small participant pool. Here, we show that concerns about non-naivete on MTurk are due less to the MTurk platform itself and more to the way researchers use the platform. Specifically, we find that there are at least 250,000 MTurk workers worldwide and that a large majority of US workers are new to the platform each year and therefore relatively inexperienced as research participants. We describe how inexperienced workers are excluded from studies, in part, because of the worker reputation qualifications researchers commonly use. Then, we propose and evaluate an alternative approach to sampling on MTurk that allows researchers to access inexperienced participants without sacrificing data quality. We recommend that in some cases researchers should limit the number of highly experienced workers allowed in their study by excluding these workers or by stratifying sample recruitment based on worker experience levels. We discuss the trade-offs of different sampling practices on MTurk and describe how the above sampling strategies can help researchers harness the vast and largely untapped potential of the Mechanical Turk participant pool.

## Introduction

In less than ten years, Amazon’s Mechanical Turk (MTurk) has gone from novel to normal as a source of participants within academic research [1]. Due to a glut of quick and affordable data, researchers from across the academy have all turned to MTurk for research participants [2–5]. Today, hundreds of peer-reviewed papers are published each year with data collected from MTurk, including, within some fields, a majority of articles in top journals [3–4, 6–7]. More than just a supplement to traditional subject pools, MTurk has become an invaluable source of quality data for scientists interested in learning about human behavior or developing new technology [8–9].

Although the use of MTurk has become ubiquitous, some researchers have questioned its suitability as a source of research participants. In particular, one question raised repeatedly in recent years is that of whether MTurk is an overused resource. Along with the knowledge that many researchers rely on MTurk, studies have shown that the average lab samples from a small subset of workers [10], and an even smaller group of “superworkers” completes most studies [11–12]. Combined, these issues amount to a general concern that MTurk participants are too familiar with manipulations and measures common in the social sciences. Thus, as sometimes happens with shared resources, there is a growing sense that MTurk is becoming overused.

Here, we argue this view of MTurk is wrong. Counter to current thinking, we suggest participant non-naivete on MTurk is driven less by any aspect of the MTurk platform itself and more by the way researchers use the platform. To support this argument, we summarize the concerns researchers have raised regarding non-naivete on MTurk, introduce data that clarifies the size of the MTurk worker pool and the prevalence of superworkers, and then describe how current sampling practices exacerbate non-naivete. Afterward, we propose and evaluate an alternative approach to sampling on MTurk that overcomes the problem of participant non-naivete without sacrificing data quality. We conclude by discussing the trade-offs between different sampling practices and when researchers might want to choose one over the other.

### Concerns about the MTurk worker pool

When it comes to non-naivete on MTurk, researchers have voiced concern about three related issues: 1) the size of the worker pool, 2) the problem of superworkers, and 3) workers’ repeated exposure to common experimental measures and manipulations. Repeatedly exposing the same group of participants to the same or similar study materials can affect participant behavior and the conclusions researchers draw from data in several ways. Most obviously, practice may improve performance on some tasks [13]. For example, experienced MTurk workers score significantly higher on the Cognitive Reflection Task (CRT) than less experienced participants in other online platforms [14–15],. Less obviously, repeated exposure to the same measures may cause participants to think harder about some tasks, think less about other tasks (due to boredom), or draw connections between the conditions of between-subjects experiments, either based on memory from previous exposure or debriefing after similar studies. Indeed, there are several theoretical reasons for researchers to be concerned about the effect of non-naivete [12], regardless of whether previous exposure helps or hinders participants within any specific study.

Despite theoretical concerns, studies demonstrating that prior exposure actually influences participant behavior on MTurk are rare. In fact, large-scale attempts to compare experimental effects on MTurk with those obtained from nationally representative samples indicate that most experimental findings replicate on MTurk [16–17]. Furthermore, studies comparing attentiveness and data quality on MTurk with other sources of research participants generally find MTurk workers perform well, passing attention checks [18], scoring high on various measures of reliability [14, 19], and providing quality data on demanding measures like reaction time tasks [20]. There are now more than enough studies assessing data quality on MTurk to fairly say that MTurk is a source of quality data. Even so, non-naivete can lower effect size estimates in at least some studies [13], meaning there are times researchers will want to sample naive participants from MTurk. We explore whether it is feasible to sample naive workers from MTurk by discussing each concern raised about participant non-naivete below.

#### How big is the MTurk worker pool?

Researchers have repeatedly tried to answer the question of how many workers are on MTurk. The answer has important implications for research, but because Amazon does not provide such information obtaining an accurate answer is difficult. What is clear from published papers is that even though Amazon advertises more than 500,000 registered workers the number of active workers is considerably smaller [10, 21–22]. Previous studies using capture-recapture analysis have estimated, on the low end, that the average lab has access to less than 10,000 workers in any three-month span [10]. Other estimates place the total number of workers at around 100,000 [21]. While these estimates are informative, the methods used to obtain them result in a wide margin of error.

We report on the size of the MTurk worker pool using observed participation rates from the TurkPrime database. TurkPrime is an independent company that makes running studies on MTurk easier for researchers by simplifying study setup and execution [23]. Since 2015, TurkPrime has recorded metadata from over 100,000 studies run on its platform. Using this data to examine worker participation rates allows us to give a concrete number on the lower bound of the MTurk population. In addition, participation rates allow us to provide a detailed analysis of how often new workers join the platform, a factor that may ultimately be more important for mitigating non-naivete than the total number of workers.

TurkPrime’s metadata showed that there were 250,810 workers worldwide who completed at least one HIT (Human Intelligence Task) posted through TurkPrime; more than 226,500 of these workers were based in the US. Our numbers indicate a lower bound of the MTurk population (i.e., there are *at least* as many workers as we report) because workers who exist on MTurk but do not take HITs through TurkPrime are not counted in our query.

**Fig 1:**
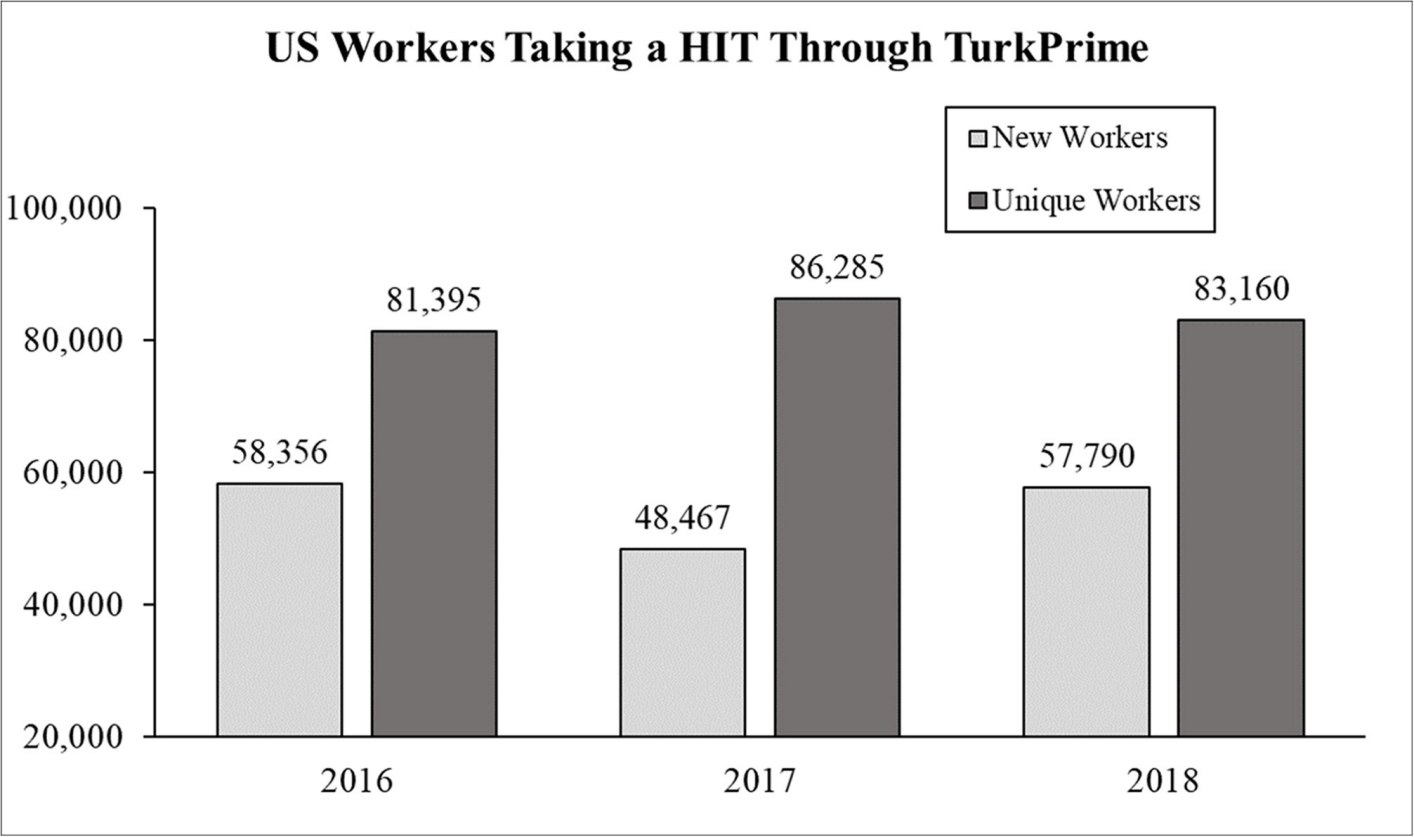
The number of new and unique US workers taking a HIT posted through TurkPrime across years.

**Fig 2:**
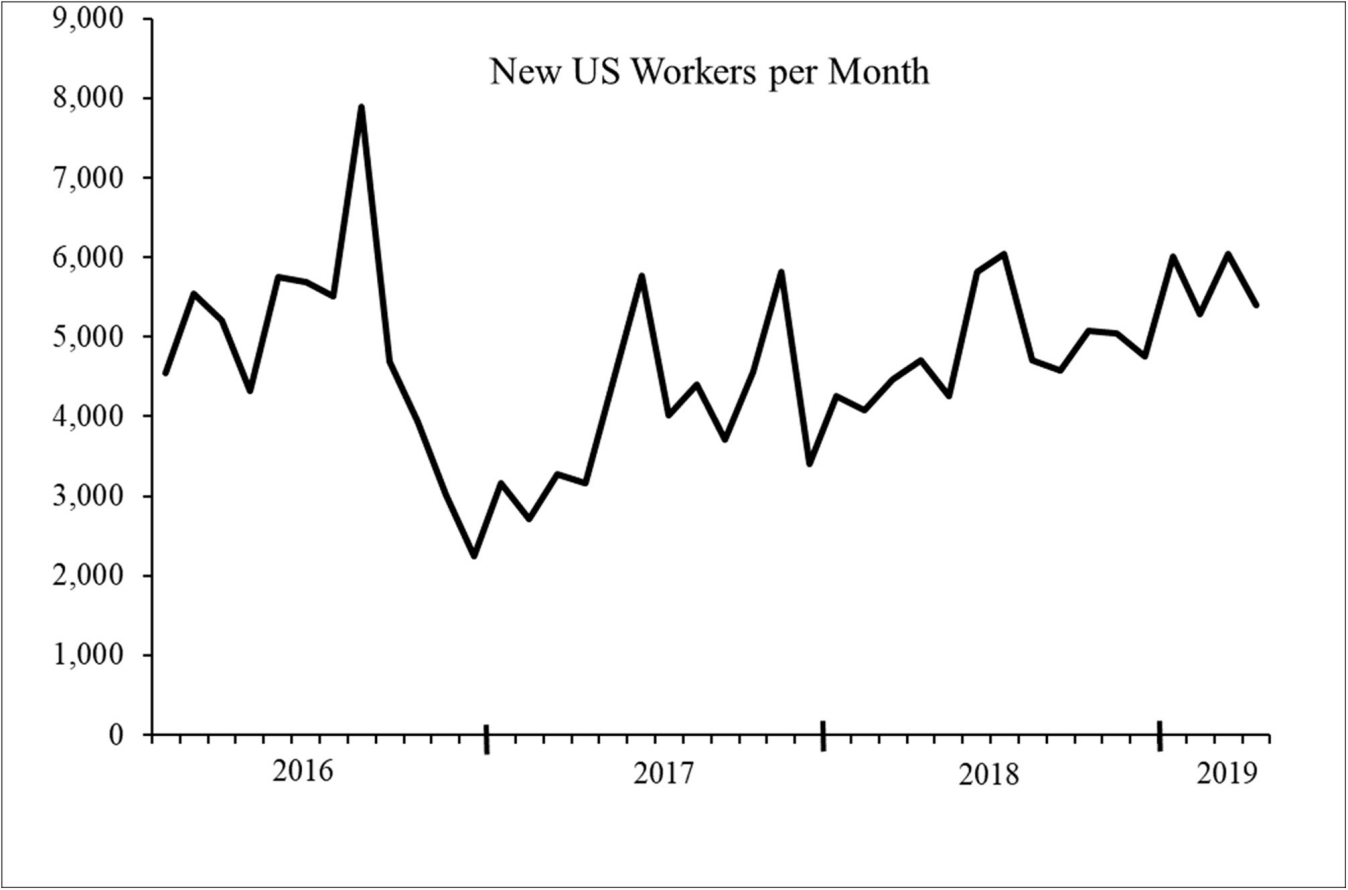
The number of new US workers per month from January, 2016—April, 2019. On average 4,683 new workers joined the pool each month.

To assess how often new workers join the platform, we examined data from January, 2016 through April, 2019 and focused exclusively on workers within the US. We focused on US workers during these years because: a) a majority of researchers are interested in sampling US participants, and b) TurkPrime data before 2016 is limited based on the number of users who ran studies. As shown in Fig 1, the total number of US workers active in any given year remained relatively stable over time. From 2016 to 2018, there were approximately 80 to 85 thousand US MTurk workers per year. Also shown in Fig 1 is the number of new US workers joining MTurk. Within any given year more than half of US MTurk workers were new to the platform. In addition, in each month from 2016 through April 2019 there were between 15,000 and 30,000 US workers taking at least one HIT and approximately 4,600 new workers joining the pool each month (Fig 2). While previous research has estimated that the average lab samples from about 7,300 workers in any three-month period [10], our findings show there are at least double this number of workers active in any single month, meaning there are thousands of workers on MTurk that researchers are not reaching. Altogether, our data show there are more workers taking HITs on MTurk than previously estimated, and more importantly, that there are thousands of new workers joining the pool each month. Indeed, the number of new workers joining the pool in any two-month period typically exceeds previous estimates about the size of the entire accessible worker pool [10].

#### How severe is the “superworker” problem?

The number of workers on MTurk is just one factor that contributes to non-naivete. Another, well-documented source of non-naivete is the “superworker” problem [11–12]. Superworkers are workers who by virtue of being very active on the platform complete more HITs than their share of the MTurk population. For example, previous research has suggested that 20% of workers may complete 80% of HITs [24].

**Fig 3:**
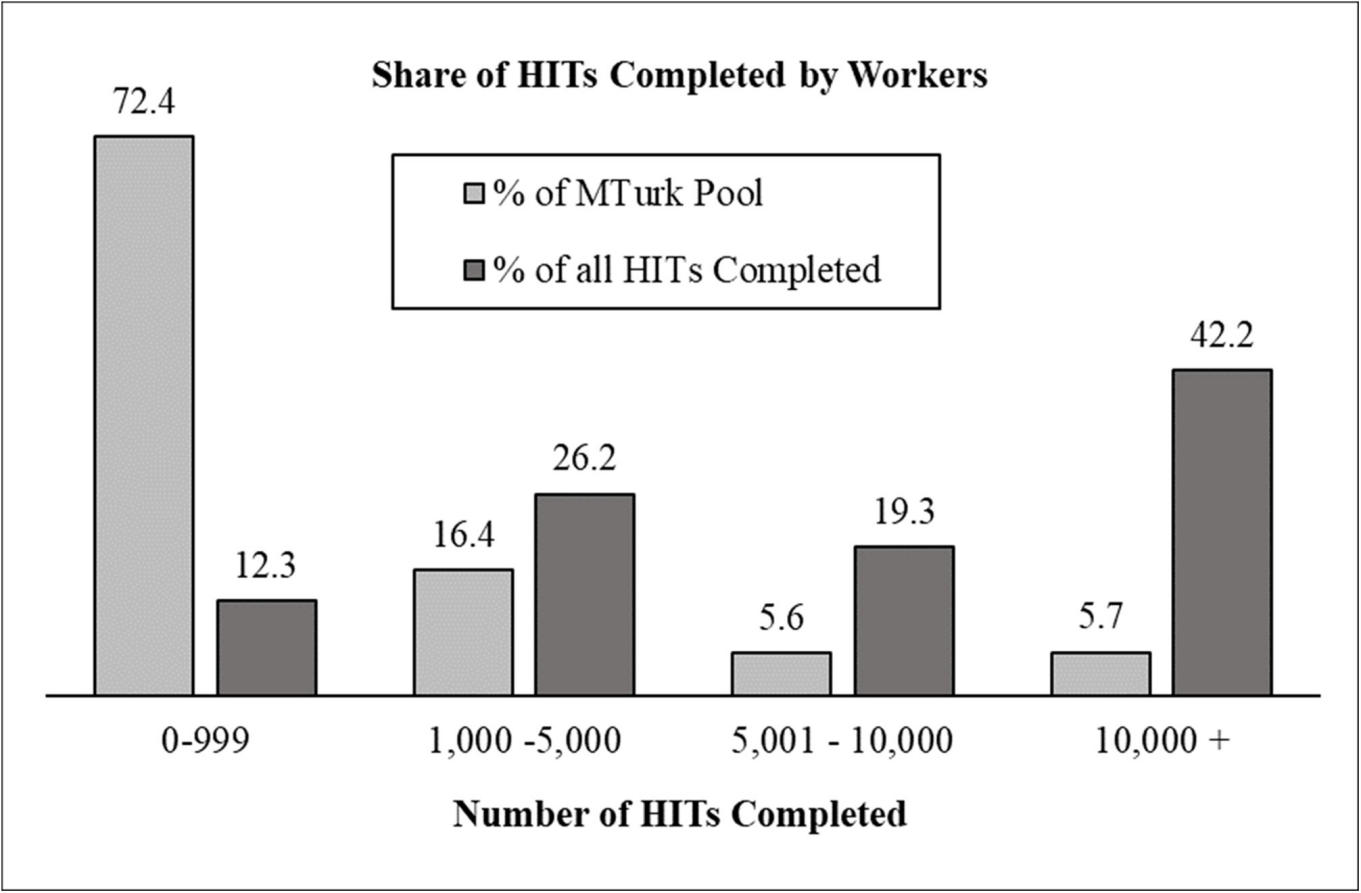
Percent of MTurk workers who fall into each experience group and the share of HITs completed by each group.

To assess the superworker problem in detail, we examined workers’ HIT completion history from MTurk. To do so, we searched for the study with the highest approval and HIT completion requirement (e.g., 500 HITs completed) each worker had completed. We then created a graph depicting workers’ activity level as a function of: a) the percentage of all HITs completed on TurkPrime, and b) the percentage of all workers. To create this graph, we divided workers into four groups with varying levels of experience. As shown in Fig 3, the largest group of workers were those with fewer than 1,000 HITs completed. Collectively, this group made up 72.4% of the entire population. However, even though the overwhelming majority of workers had fewer than 1,000 HITs completed this group took just 12.3% of all HITs.

More specific to the issue of superworkers, the right half of Fig 3 shows that just 11.3% of workers had more than 5,000 HITs completed, and that this group took nearly two-thirds of all HITs. Equally striking, the most active 5.7% of workers completed 42.2% of all HITs. Thus, from these data, it is clear the superworker problem is even more extreme than previously thought. A small group of workers on MTurk complete far more HITs than researchers realize. Across the three years we examined, 5.7% of the approximately 85,000 US workers—just 4,845 people on average—made up close to half of the participants in MTurk studies run each year.

#### Why are most samples comprised of non-naive participants?

The data reported so far appear to present a paradox. On the one hand, MTurk has several hundred thousand workers. On the other hand, just a sliver of those workers constitutes a majority of the participants in almost all studies. How is this possible? There are at least three compatible explanations. First, experienced workers may wind up in most studies because they spend more time on MTurk than less experienced workers. If so, this may be because the most experienced and most active workers have a strong commitment to earning money on MTurk. A second possibility is that experienced workers use technology and tools inexperienced workers do not know about and which allow experienced workers to quickly grab desirable HITs [25]. If this explanation is true, it suggests inexperienced workers are crowded out of most HITs. Finally, a third, entirely overlooked possibility is that the way researchers sample from MTurk exacerbates non-naivete by making a large chunk of inexperienced workers ineligible for most studies. If this explanation is true, it suggests inexperienced workers are locked out of HITs by the sampling practices researchers use. While all three explanations can be true at the same time, we believe the biggest driver of non-naivete is the sampling practices used by researchers. In the space below, we describe how current sampling practices that were originally adopted to maintain data quality now contribute to non-naivete. Then, we propose and evaluate an alternative method of sampling from MTurk that avoids the problem of participant non-naivete without sacrificing data quality.

### Current sampling practices on MTurk

All behavioral science studies run online share at least one thing in common: concern from researchers about data quality. From the first online studies in the 1990’s [26] to the thousands of projects run on Mechanical Turk today, researchers often stand somewhere between mildly concerned to deeply suspicious about whether participants are who they say they are and whether they are paying attention while taking studies. Despite this concern, numerous investigations have demonstrated that MTurk workers produce data comparable to if not better than other, more traditional sources of participants like student samples [18, 27]. As several researchers have noted, part of the reason MTurk workers produce quality data is because of Mechanical Turk’s reputation mechanism [28]. Concern about having their work rejected—and their reputation damaged—leads most workers to give a good effort.

Over time, researchers’ concern about data quality has led most to adopt the practice of selectively sampling workers with an already established reputation. Consistent with the results of Peer et al., [28], most researchers have adopted the criteria of a 95% approval rating and at least 100 HITs completed [28–31]. However, these reputation qualifications present a problem for two reasons. First, a fine-grained look at the data on workers’ HIT completion history indicates that close to 35% of MTurk workers have completed fewer than 100 HITs. Under current sampling practices, these workers are locked out of studies. Second, as the data in Fig 3 show, workers with fewer than 1,000 previous HITs completed take a small fraction of overall studies. This means that, without knowing, researchers are excluding the most naive workers and continuously launching studies to the same small group of experienced workers because of concerns about data quality. This practice exacerbates non-naivete. And importantly, there are reasons to question whether this trade-off is worth making.

Perhaps the most important reason to question whether it is necessary to selectively sample high reputation workers in order to collect quality data is that there may be few low reputation workers on MTurk. Peer et al., [28] suggested this possibility based on the difficulty they had recruiting workers with a low reputation. For example, Peer et al., (see Study 2 in [28]) tried to recruit workers with an approval rating below 90% and over the course of 10 days received only 30 responses. One reason for this may be that most workers hold inflated reputations. Peer et al., [28] suggested this could occur, stating that if requesters approve more HITs than they should, worker reputations would be less indicative of data quality (p. 1031).

From our perspective, there are two potentially faulty assumptions behind reputation qualifications as they are currently used. First, there seems to be an assumption that without reputation qualifications a significant number of low reputation workers will take a study. However, exclusively targeting low reputation workers (as Peer and colleagues did) is not the same thing as leaving a study open to workers who may have a low reputation. Because there very well may be few low reputation workers on MTurk, a study run without standard qualifications may not collect many workers with an approval rating below 95%. Second, there seems to be an assumption that the data obtained in studies using standard qualifications will be better than that obtained in studies not using standard worker qualifications. However, there is no direct evidence for this idea. Thus, we tested both assumptions by running experiments where we varied worker qualifications and examined worker naivete along with several measures of data quality.

Our studies had two goals. First, we sought to ascertain whether dropping the requirements that workers have 100 prior HITs completed and a 95% approval rating would affect sample composition. In line with the idea that there may not be many workers with a low reputation on MTurk, we expected the majority of workers in a sample gathered with no reputation qualifications to look like a standard sample (i.e., workers who have completed 100 prior HITs and have a 95% approval rating). Second, we sought to use the worker HIT completion qualification to restrict the participation of highly active workers—the opposite of how this qualification is currently used. We hypothesized that because all workers have a motivation to avoid rejections, using the HIT completion qualification to sample inexperienced workers would result in a sample of naïve participants who provide high quality data.

## Overview

We conducted two studies to investigate whether it is possible to sample inexperienced workers from MTurk and whether these workers provide quality data. In both studies, we examined data quality among groups of workers sampled with different reputation qualifications. In Study 1, we sampled three groups of workers: one using standard reputation qualifications (standard sample), one without any qualifications (open sample), and one where we used the HIT completion qualification to target inexperienced workers (inexperienced sample). In Study 2, we omitted the open sample and only gathered data from a standard sample and an inexperienced sample. In both studies, the standard sample served as a baseline and represented the data quality commonly obtained by researchers using MTurk. We expected that the standard and open samples in Study 1 would look nearly identical in terms of both worker reputation and data quality. More specifically, even though anyone was eligible to participate in the open sample, we expected that nearly the entire sample would consist of workers with a 95% approval rating and at least 1,000 prior HITs completed. Finally, the most important sample in both studies, the inexperienced sample consisted of workers with *less than* 50 HITs completed. The inexperienced sample allowed us to explore whether researchers can gather quality data from naive participants by changing sampling practices in order to target the approximately 70% of inexperienced workers who are currently excluded by standard reputation qualifications.

Participants in both studies completed several measures that we used to examine data quality. All participants completed a common personality questionnaire, three short experiments, the Cognitive Reflection Task, and attention check questions. We assessed data quality by examining the reliability of participant’s responses to the personality questionnaire, the replicability of well-established experimental manipulations, and performance on the attention check questions. We used the cognitive reflection task and questions asking participants whether they had ever seen the experimental manipulations before as indicators of non-naivete. We expected to find that inexperienced participants would provide quality data comparable to that of a standard sample across indicators of consistency on the BFI and effect sizes on the experimental manipulations.

### Data, materials, and online resources

All materials, data, and analysis scripts for the studies reported in this paper are available online at [https://osf.io/vsm5b/?view_only=423369ebcf604cd2b6d73b14e601908d].

### Reporting and ethical approval

We report how we determined sample size, all data exclusions, all manipulations, and all measures in the study with two exceptions. First, in both studies our survey instruments included several measures we have used in past research but that are not relevant to data quality in the current study. These measures primarily assess people’s attitudes toward a variety of political issues. Second, we asked participants some demographic questions that we also do not report in this paper. We omitted these measures from this report because they were not relevant to our methodological point. The data files are freely available for researchers interested in looking at unreported measures.

The research reported here was approved by IntegReview, an independent institutional review board that reviews research involving human subjects. The protocol number is TurkPrime 002.

## Study 1

### Method

#### Participants and procedure

We aimed to collect data from 750 people—250 in each of our three samples (standard, open, inexperienced). We expected the study to take about 15 minutes and paid each participant $1.00. Although we did not conduct a formal power analysis, we aimed to recruit large samples in line with past work examining data quality on MTurk [1, 28].

The final dataset included 768 responses. There were more responses than participants we aimed to collect data from because two participants entered the study more than once and 34 people dropped out of the study early. We retained data from all participants who completed all our measures of data quality (*n* = 758). This cutoff resulted in removing incomplete responses from the two people who entered the study more than once and eight people who completed less than 35% of the study. After exclusions, the sample included nearly equal numbers of men (*n* = 375) and women (*n* = 350), and the average age was 33.9 years (*SD* = 10.17) (see Table 1 for detailed demographic information).

**Table 1:** Basic demographics for the standard, open, and inexperienced samples.

	Sample		
	Standard	Open	Inexperienced
Annual household income			
< 20k	12.2	18.8	12.8
20-39k	26.7	25.8	18.4
40-59k	25.1	17.5	23.1
60-79k	14.9	15.8	15.4
80-99k	8.6	8.8	10.3
>100k	12.5	13.3	20.1
Marital status			
Married	31.8	32.8	39.1
Separated	1.2	0.8	1.3
Widowed	-	0.4	-
Divorced	9.0	8.0	8.5
Never married	58.0	58.0	51.1
Children			
Yes	29.8	34.3	38.7
Race			
White	75.8	79.9	80.8
Black	9.1	8.4	6.0
Asian	8.7	6.7	4.3
Biracial	3.2	1.3	2.1
Other	3.2	3.8	6.8
Highest Degree			
No college degree	40.8	37.3	36.2
College degree	51.4	54.4	49.8
Post-college degree	7.8	8.3	14.0
Political party			
Republican	20.2	18.8	20.4
Democrat	48.2	45.8	30.6
Independent	26.9	28.7	29.8
Other	3.2	1.3	4.3
No preference	1.6	5.4	14.9
Religion			
Buddhist	-	0.4	1.3
Christian	38.4	40.8	53.8
Muslim	2.0	0.4	-
Jewish	1.2	2.5	-
Hindu	0.4	-	-
Agnostic	20.0	25.4	15.0
Atheist	26.7	19.6	14.5
Other	8.2	7.9	9.4
Prefer not to say	1.6	2.9	6.0

*Note*: College degree = 2 or 4 year degree

To recruit participants, we created three separate studies on MTurk and varied the worker qualifications for each. All three studies were setup and managed using the TurkPrime platform [23]. In the first study (Standard), we used standard worker qualifications of at least a 95% approval rating and more than 100 HITs completed. In the second study (Open), we used no qualifications, meaning the study was open to all workers on MTurk. Finally, in the third study (Inexperienced), we required workers to be inexperienced by setting the qualification requirement to *less than* 50 HITs completed. Data collection for all three studies started at the same time and ended after approximately one day (standard = 25 hours, open = 22 hours, inexperienced = 25 hours). After all three studies ended, we used the TurkPrime database to query workers’ approval rating and number of HITs completed in the open sample.

Participants completed the Asian Disease experiment, Mt. Everest experiment, Trolley Dilemma experiment, Big Five Personality Inventory (BFI), Cognitive Reflection Test (CRT), and demographic questions. Each experimental manipulation—Asian Disease, Mt. Everest, Trolley Dilemma—had two conditions and participants were randomly assigned to conditions. The order of the experimental manipulations, the BFI, and the CRT was randomized across participants. After participants completed all tasks, they answered demographic questions. We included four attention check questions at various points in the survey—two in the BFI and two in the demographics section.

### Measures

#### Asian disease problem

The Asian Disease problem is a classic framing effect [32]. In it, people are asked to imagine the US is preparing for an outbreak of disease that is expected to kill 600 people. Then, participants are asked to choose between two logically identical courses of action, framed in terms of either gains (lives saved) or losses (lives lost). People in the gains condition are asked whether they would adopt a program that is certain to save 200 people or a program in which there is a one-third probability that 600 people will be saved and a two-thirds probability that no one will be saved. People assigned to the loss condition are asked whether they would adopt a program in which it is certain that 400 people will die or a program where there is a one-third probability nobody will die and a two-thirds probability that 600 people will die. Studies have repeatedly found that when outcomes are framed in terms of lives saved, people prefer the certain option, but when outcomes are framed in terms of lives lost, people prefer the uncertain option.

We examined whether this effect replicated among the standard, open, and inexperienced samples, and whether the effect sizes were similar across groups. To measure naïveté, we asked participants whether they had ever responded to the problem previously. For this task and all other tasks, the question about naivete was presented after participants responded to the measure.

#### Mt. Everest experiment

The Mt. Everest experiment is a classic anchoring effect [33]. In it, people are asked to estimate the height of Mt. Everest after being randomly assigned to a low or high anchor condition. In the low anchor condition, people are asked whether Mt. Everest is greater or less than 2,000 feet in height. In the high anchor condition, people were asked whether Mt. Everest is greater or less than 45,000 feet in height. Finally, people are asked to guess the height of Mt. Everest. Jacowitz and Kahneman [33] found that people exposed to the high anchor tend to provide larger estimates than people exposed to the low anchor.

#### Trolley dilemma experiment

Based on a thought experiment by Thomson [34], the Trolley Dilemma asks people whether they would sacrifice one person to save the lives of five others (1*—Definitely not*, 2*—Probably not*, 3*—Probably yes*, 4—*Definitely yes*). Participants were randomly assigned to one of two versions of the dilemma. In the classic version, people were asked to imagine they are driving a trolley with failed brakes, which will collide with and kill five people. Participants can save the people by turning the trolley onto another track, but this would result in killing one person. In the footbridge version, people were asked to imagine that a trolley with failed brakes is heading toward five people. Participants can save the people by pushing an innocent bystander in front of the train. Numerous studies have found that peoples are more willing to sacrifice one life in order to save five when doing so requires turning the train than when doing so involves pushing a man off the bridge [35].

#### Big-five inventory

The BFI personality questionnaire [36] consists of 44 short declarative statements such as “Is talkative.” People indicate whether each statement applies to them (1—*strongly agree* to 5—*strongly disagree*). Approximately half of the items for each trait were reverse coded. We also added ten items that were direct antonyms of original items. For example, “tends to be organized” was reversed to be “tends to be disorganized.” These items were used to examine the consistency of participants’ responses using the Squared Discrepancy Procedure (SDP).

#### Squared discrepancy procedure

The Squared Discrepancy Procedure is a statistical approach for analyzing individual-level response-consistency to reverse scored Likert items [37]. For the BFI, the discrepancy between each item and its reversed form can range between zero, no discrepancy (e.g., answering 5 “strongly disagree” for being organized, and 1 “strongly agree” to being disorganized) and a maximum discrepancy score of four (e.g., answering 1 “strongly agree” to both being organized and being disorganized). In the procedure, discrepancies between reversed items are squared to place a greater emphasis on highly inconsistent responses than slightly inconsistent responses. Next, the sum of squared discrepancy scores from all ten pairs of questions is converted to a percentage that ranges from 0% to 100% and then reversed so that a score of 0% indicates *maximally inconsistent performance* and a score of 100% indicates *maximally consistent performance*. A Monte Carlo simulation indicated that truly random responses produce an SDS of 70 (*SD* = 7) [37]. We defined consistent responders as those who demonstrate consistency scores greater than two standard deviations above what would be expected by a random responder.

#### Cognitive reflection test

The Cognitive Reflection Test (CRT) consists of three questions that measure the tendency to provide an intuitively compelling, but incorrect, response over a reflective, yet correct response [38].

#### Attention check questions

We used four attention check questions to assess people’s attentiveness. The first two questions were inserted in the BFI and took the same form as the other BFI questions. The first attention check question read, “is someone who reads the questions on surveys,” and the second question read, “is not reading the questions in this survey.” The response options for these questions were the same as for the other BFI items (1 = *Disagree strongly*, 5 = *Agree strongly*). We scored responses as correct if participants answered agree strongly to the first question and disagree strongly to the second question.

Two other attention check questions were embedded in the demographics section. The first question read, “I am not reading the questions in this survey,” and had five response options ranging from (1 = *Disagree strongly*) to (5 = *Agree strongly*). The second question read, “Please select ‘Satisfied’ on the scale (second from the left): This item is for verification purposes,” and had five response options ranging from (1 = *Very satisfied*) to (5 = *Not at all satisfied*). We scored these questions as correct if participants answered disagree strongly to the first question and satisfied to the second question.

#### Demographics

After completing all other measures, we asked people about their gender, race, age, level of education, marital status, political affiliation, religion, and household income. These questions were taken from the ANES survey [39].

## Results

### Analytic approach

#### Determining approval ratings and HIT completion history in the open sample

MTurk does not provide workers’ approval rating or HIT completion history. As a result, we inferred the qualifications of each worker in the open sample by querying TurkPrime’s database to find the maximum qualification criteria each worker had met in previous HITs. We reasoned that a worker who qualified for a study with a 95% approval rating and 1,000 HITs completed must have at least a 95% approval rating and at least 1,000 HITs completed. We refer to the qualification criteria we queried as each worker’s verified approval rating and verified HIT completion history.

#### Experimental manipulations

We examined whether experimental effects replicated across samples, and report effect sizes of the various samples. For the Mt. Everest experiment, which has a continuous dependent variable, we conducted linear regression. For the Asian Disease and Trolley Dilemma experiments we used a similar approach but conducted logistic regression to accommodate the binary dependent variables.

#### BFI

We computed alpha reliability coefficients for each dimension of the BFI and then used the coefficients to compare group-level reliability. We also used the Squared Discrepancy Procedure [37] to compute individual-level measures of reliability for each participant.

#### CRT

We analyzed the cognitive reflection test results with linear regression.

### Approval rating and HIT completion history in the open sample

**Fig 4:**
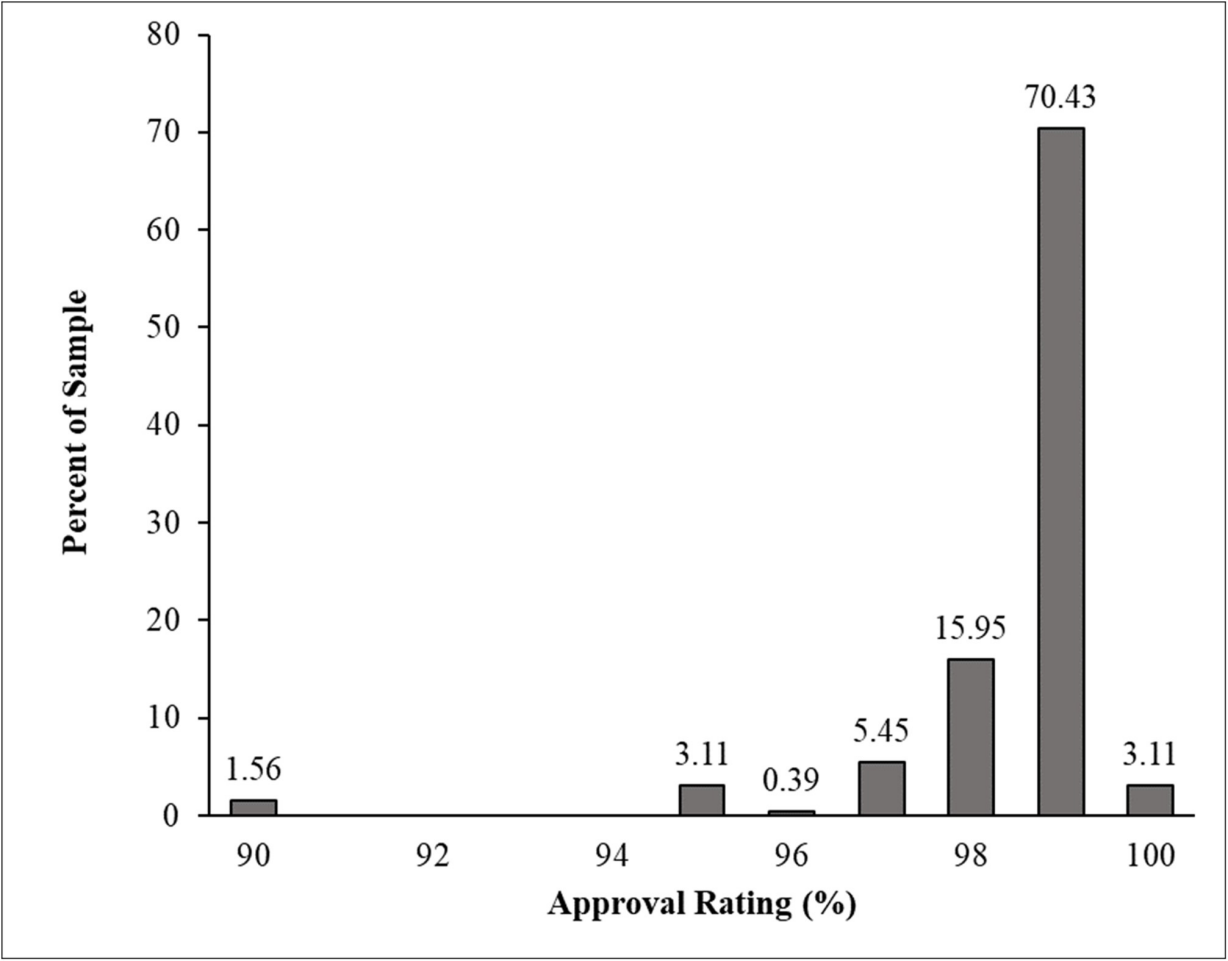
Verified approval rating for workers in the open sample.

One assumption behind the use of standard worker qualifications is that without such qualifications many low reputation workers who offer poor data may take a study. In our open sample, we found four workers out of 250 (1.93%) with a verified approval rate of 90%; all other workers had a verified approval rate of 95% or higher (Fig 4).

**Fig 5:**
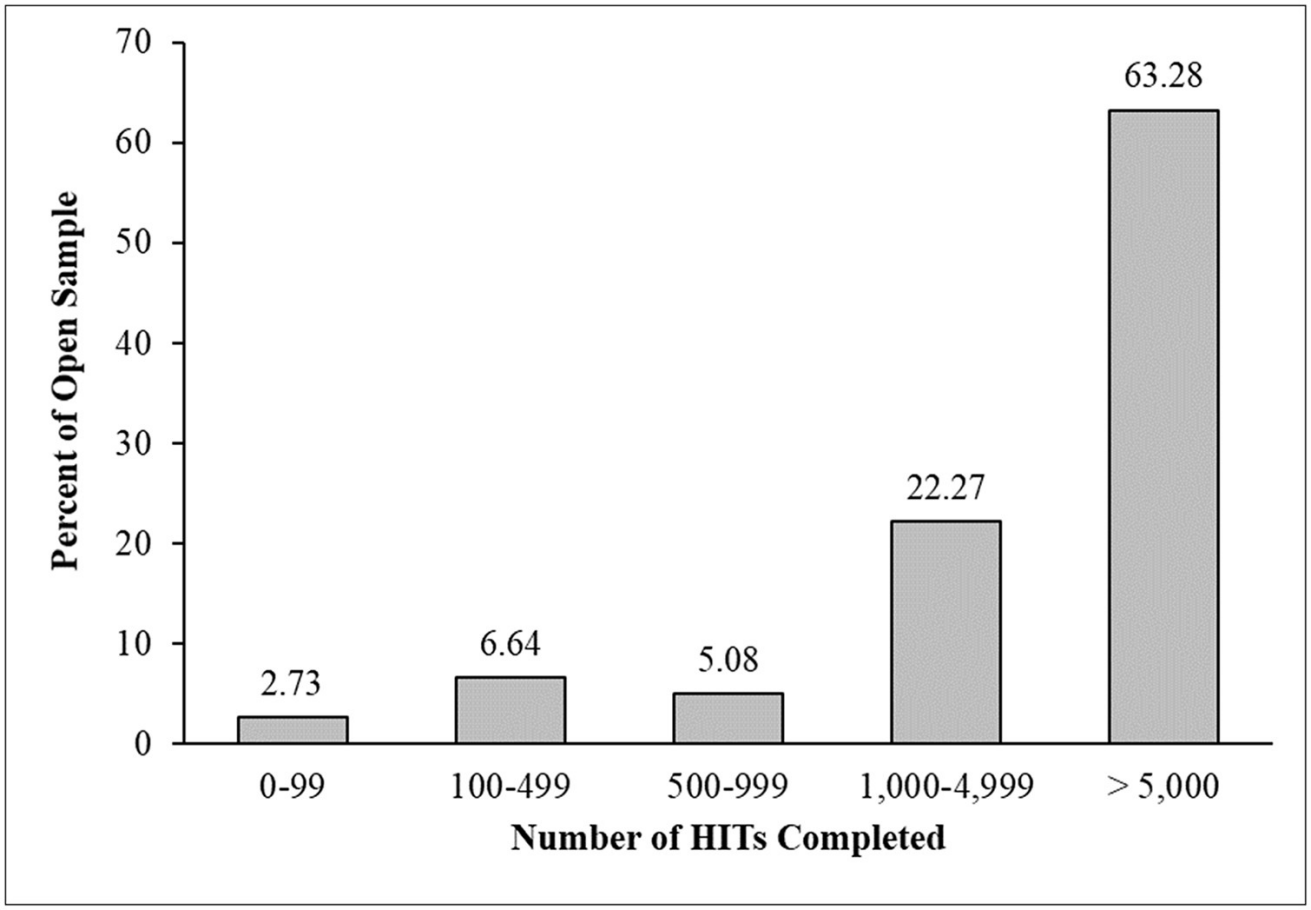
Verified HIT completion history for workers in the open sample.

Regarding participant experience in the open sample, we found just seven workers had completed fewer than 100 HITs. Almost all workers in the open sample (97.3%) had a verified HIT completion of over 100, with 85% over 1,000 HITs, and the majority (63.28%) having a verified HIT completion of 5,000 or more (Fig 5). The presence of so many experienced workers with a high approval rating in a study open to all workers strongly suggests there is little reason to fear that omitting worker qualifications will result in a sample of low-reputation workers. Furthermore, the composition of the open sample strongly supports our prediction that there is little difference in the workers that researchers sample when using standard qualification criteria and when not using these criteria.

### Data quality

We examined participants’ performance on attention checks, the time it took to complete the study, and self-reported rates of prior exposure to each task (i.e., naivete) as issues that may affect data quality.

#### Attention check questions

Table 2 shows that a similar percentage of participants in each sample passed all four attention check questions. Even though about 20% of participants in each sample missed at least one question, roughly 90% of participants answered three out of four questions correctly. In addition, for the question with the clearest scoring criteria—“please select “Satisfied” for verification purposes”—which appeared near the end of the survey, we found that all three groups passed the question at a high rate (98.8% in the standard sample, 98.3% in the open sample, 95.7% in the inexperienced sample).

**Table 2:** The percentage of participants who passed the attention check questions in Study 1.

Sample	Passed all ACQs	Number of ACQs failed			
		1	2	3	4
Standard	81.2	11.1	5.0	0.8	1.9
Open	78.8	13.5	6.9	0	0.8
Inexperienced	78.6	8.4	8.4	2.7	1.9

*Note*: ACQ = attention check question

#### Completion time

We used the start and stop time of each participant’s survey as recorded by Qualtrics to examine how long it took participants to complete the study. We excluded one person whose timing data said it took 280 minutes to complete the study from the completion time analysis. Overall, people completed the study in 14.44 (*SD* = 7.43) minutes on average. However, the time to complete the study significantly differed between groups, *F*(2,754) = 42.64, *p* < .001. Sheffe-corrected post-hoc comparisons showed that people in the inexperienced sample took significantly longer to complete the study in minutes (*M* = 17.75, *SD* = 7.00) than those in both the standard (*M* = 12.67, *SD* = 7.21), *p* < .001, and open samples (*M* = 12.82, *SD* = 6.94), *p* < .001. People in the standard and open samples took about the same amount of time, *p* = .97.

#### Exposure rates

As expected, people reported more prior exposure to the materials in the standard and open samples than in the inexperienced sample (see Table 3). Differences in prior exposure were particularly striking for the CRT, where close to 75% of participants in the standard and open samples reported having previously seen the task, while only about one-fourth of participants in the inexperienced sample did so. Due to a programming error, non-naivete was only measured for the classic version of the trolley dilemma. Also, of note, all participants reported low previous exposure to the Mt. Everest anchoring task.

**Table 3:** The percentage of participants reporting prior exposure to the experimental measures in Study 1.

Sample	Experimental Task			
	Cognitive Reflection Task	Trolley Dilemma	Asian Disease	Mt. Everest
Standard	75.0	63.8	32.3	5.6
Open	71.4	53.9	29.6	4.8
Inexperienced	27.6	14.0	2.3	3.1

*Note*: Percentages in the trolley dilemma column are based only on respondents assigned to the classic condition.

#### Squared discrepancy scores

As a reminder, the squared discrepancy score is a measure of individual level response consistency to 10 pairs of antonymous statements embedded within the BFI. Previous research has established that a score of 70 corresponds to completely random responding and that a score of 84—two standard deviations above chance performance—indicates attentive responding [37]. The consistency scores of participants in the three groups are presented in Table 4. In Table 4, z scores correspond to how many standard deviations above chance participants in each group scored. Participants whose SDS scores were below a z-score of 2 were responding inconsistently and not significantly better than chance [37]. Higher z scores represent more consistent responding.

**Table 4:** Comparison of squared discrepancy scores across sample groups in Study 1.

Z-scores	Below 1		Between 1–2		Between 2–3		Between 3–4		Above 4	
Sample	N	% of Sample	N	% of Sample	N	% of Sample	N	% of Sample	N	% of Sample
Standard sample	12	4.7	18	7.0	35	13.7	94	36.7	97	37.9
Open sample	11	4.5	9	3.7	40	16.3	92	37.6	93	38.0
Inexperienced	1	.4	8	3.1	30	11.7	137	53.3	81	31.5

Further analyses indicated that the differences between groups were statistically significant, *F*(2, 755) = 4.31, *p* = .01. People in the inexperienced group were more consistent in their responses (*M* = 95.10, *SD* = 4.50) than people in the standard group (*M* = 93.40, *SD* = 8.02), *p* = .02 (Scheffe-corrected). In addition, people in the open group (*M* = 93.96, *SD* = 7.00) were similar in consistency to the standard group, *p* = .65, and people in the inexperienced and open groups were not significantly different, *p* = .16.

### Experimental results

#### Asian disease

Response frequencies for the Asian Disease experiment are presented in Table 5. As shown, the manipulation replicated in all cases except one: the negative frame in the standard sample. A logistic regression examining the condition by group interaction (Condition effect [% who chose program A in the Positive Frame—% who chose program A in the Negative Frame] standard = 29.1%, open = 26.1%, inexperienced = 31.%) was not statistically significant when comparing the inexperienced sample to the standard sample (Logistic regression OR = 0.98, *p* = .95) or the inexperienced sample to the open sample (Logistic regression OR = 0.81, *p* = .57).

**Table 5:** The percentage of participants choosing each response option in the Asian Disease experiment in Study 1.

Sample	Positive Frame		Negative Frame	
	Response A	Response B	Response A	Response B
Standard	77.2*	22.8	48.1	51.9
Open	71.2*	28.8	45.0*	55.0
Inexperienced	60.9*	39.1	29.5*	70.5

Note:

* Indicates a statistically significant χ^2^ at the *p* < .05 level, relative to a H_o_ value of 50%.

#### Mt. Everest

Participants typed their estimate for the height of Mt. Everest into the computer. Five participants provided estimates that were greater than five standard deviations from the mean. We analyzed the data without making any adjustments and then after winsorizing outliers.

**Fig 6:**
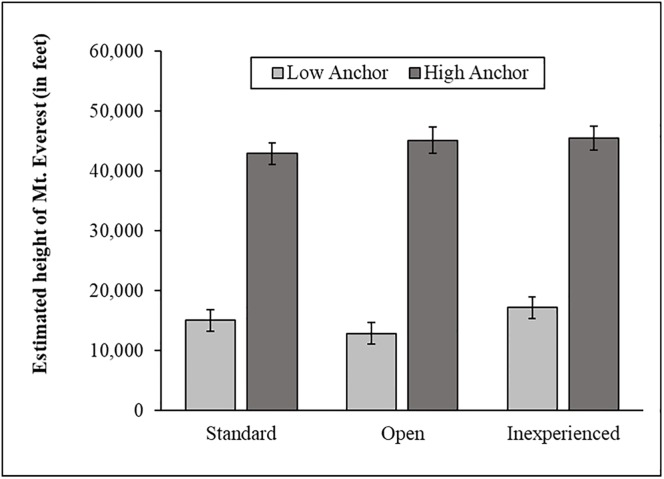
The anchoring manipulation across all three groups in Study 1.

When examining all data, the anchoring manipulation worked in all three groups: standard *t*(254) = 9.10, *p* < .001, *d* = 1.14, 95% CI [0.87, 1.40], open, *t*(243) = 9.31, *p* < .001, *d* = 1.19, 95% CI [0.92, 1.46], inexperienced, *t*(255) = 5.88, *p* < .001, *d* = 0.74, 95% CI [0.48, 0.99]. A regression analysis revealed a large difference in people’s estimates based on anchoring condition, *b* = 28,867, *t* = 7.79, *p* < .001, 95% CI [21,594, 36,140], but no overall difference in the estimates between groups (*p*s > .12) and no significant interactions (*p*s > .25). Winsorizing estimates greater than five standard deviations from the mean yielded similar results. The anchoring manipulation worked in all three groups: standard *t*(254) = 10.90, *p* < .001, *d* = 1.36, 95% CI [1.09, 1.63], open, *t*(243) = 11.52, *p* < .001, *d* = 1.47, 95% CI [1.19, 1.75], inexperienced, *t*(255) = 10.43, *p* < .001, *d* = 1.30, 95% CI [1.03, 1.57] (Fig 6). A regression analysis revealed a large difference in people’s estimates based on anchoring condition, *b* = 28,566, *t* = 11.68, *p* < .001, 95% CI [23,767, 33,366], but no difference in the overall estimates between groups (*p*s > .38) and no significant interactions (*p*s > .30).

#### Trolley dilemma

Response frequencies for the trolley dilemma are presented in Table 6. The magnitude of the trolley dilemma effect as measured by a condition by group interaction (Condition effect [Yes response to the Classic dilemma—Yes response to the Footbridge dilemma] standard = 48.4%, open = 39.2%, inexperienced = 73.5%) was significantly higher for the inexperienced compared to the standard (Logistic regression OR = 6.22, *p* < .001) and open samples (Logistic regression OR = 9.74, *p* < .001). Inexperienced participants produced a significantly larger effect size in the trolley dilemma experiment than participants in either of the other two groups. Effect sizes for all groups for all three experiments are presented in Table 7.

**Table 6:** The percentage of participants choosing each response option in the trolley dilemma in Study 1.

Sample	Version of Dilemma			
	Classic		Footbridge	
	No	Yes	No	Yes
Standard	26.0	74.0*	74.4*	25.6
Open	27.0	73.0*	66.2*	33.8
Inexperienced	7.8	92.2*	81.3*	18.7

Note:

* Indicates a statistically significant χ^2^ at the *p* < .05 level, relative to a H_o_ value of 50%.

**Table 7:** Effect sizes (Cohen’s d) of sample groups across experimental tasks in Study 1.

Sample	Experimental Task		
	Asian Disease (odds ratio)	Mt. Everest (Cohen’s d)	Trolley Dilemma (odds ratio)
Standard	3.65	1.47	8.27
Open	3.00	1.5	5.29
Inexperienced	3.72	1.28	51.05

#### BFI scores

Reliability coefficients are presented in Table 8. For comparison, Table 8 also presents reliability coefficients for a standard internet sample, as reported in Buhrmester et al. [1]. People in the inexperienced group were similar to the standard group in dimensions of neuroticism and extraversion, and somewhat lower in the openness, conscientiousness, and agreeableness dimensions. The reliabilities for the inexperienced group were virtually identical to the standard internet sample and in line with participants who have not seen the BFI repeatedly. Finally, the open group was virtually identical to the standard group along all dimensions.

**Table 8:** Alpha coefficients for the dimensions of the BFI in Study 1.

Dimension	Sample			
	Standard	Open	Inexperienced	Buhrmester et al., 2011
Openness	.88	.86	.79	.79
Conscientiousness	.85	.87	.77	.77
Extraversion	.89	.87	.87	.87
Agreeableness	.85	.86	.76	.77
Neuroticism	.77	.81	.74	.85

#### CRT scores

CRT scores differed significantly between the three groups, *F*(2, 754) = 25.81, *p* < .001, R^2^ = .06. The standard group (*M* = 2.01, *SD* = 1.14) answered more questions correctly than the open group (*M* = 1.74, *SD* = 1.21), *b* = -0.26, *t* = -2.55, *p* = .01, 95% CI [-0.47, -0.06]. In addition, both the standard, *b* = 0.73, *t* = 7.10, *p* < .001, 95% CI [0.53, 0.93], and open groups, *b* = 0.47, *t* = 4.47, *p* < .001, 95% CI [0.26, 0.67], answered more questions correctly than the inexperienced group (*M* = 1.28, *SD* = 1.14). These findings are in line with previous research showing that people with previous exposure to the CRT score higher than those without previous exposure [14]. Indeed, previous exposure to the CRT was positively correlated with performance (*r* = .31, *p* < .001).

### Discussion

The results of Study 1 suggest a number of important things. First, although most researchers use a 95% approval rating and at least 100 HITs completed as standard worker qualifications, these qualifications may do little to improve data quality. We found that a sample gathered with no worker qualifications looked essentially the same as a sample gathered with standard worker qualifications. Nearly all (246 of 250) of the workers in the open sample had an approval rating above 95% and more than 85% of the sample had completed more than 1,000 HITs. Perhaps more importantly, participants collected without any worker qualifications passed attention checks at a high rate, had high internal consistency scores on a personality measure, and yielded effect sizes in the expected range on three experimental manipulations. The data quality observed in the open sample and the results seen in Fig 5 indicate that there may be few workers on MTurk with a poor reputation.

Secondly, the results of Study 1 suggest that it is possible to gather quality data from inexperienced workers. Like the workers in the open sample, workers with less than 50 prior HITs completed had a high pass rate on attention check questions, responded consistently to a long personality measure, and produced effect sizes in three experimental manipulations that were consistent with previous findings (and in one case significantly larger than the standard and open samples). In addition, workers in the inexperienced sample reported less prior exposure to all experimental manipulations than workers in the standard or open samples (Table 3). On two tasks—the trolley dilemma and cognitive reflection task—there was evidence that inexperienced workers’ lack of prior exposure was important. Inexperienced workers produced a larger effect size on the trolley dilemma and scored closer to the average for people who have not seen the cognitive reflection task than did workers in the standard sample. Overall, these results suggest that researchers can collect quality data while sampling naive workers from MTurk.

To increase confidence in our results, we conducted a replication. Study 2 was conducted nearly one year after Study 1 and after the data quality scare that affected MTurk in the summer of 2018 [40]. In Study 2, we omitted the open condition because the results of Study 1 showed the group was virtually identical to the standard sample in terms of worker experience. With the exception of this change, Study 2 was exactly the same as Study 1. We expected our results to replicate on all measures.

## Study 2

### Method

#### Participants and procedure

We aimed to collect data from 300 people—150 in each sample (standard, inexperienced). We expected the study to take about 15 minutes and paid each participant $1.50. The final dataset included 313 responses. Like Study 1, there were more responses than participants we aimed to collect data from because two participants entered the study more than once and nine people dropped out of the study early. We retained data from all participants who completed all measures of data quality (*n* = 302). This cutoff resulted in removing incomplete responses from the two people who entered the study more than once and nine people who completed less than 37% of the study. After exclusions, the sample included nearly equal numbers of men (*n* = 146) and women (*n* = 155), and the average age was 34.57 years (*SD* = 10.60) (see Table 9 for detailed demographic information).

**Table 9:** Basic demographics for the standard and inexperienced samples.

	Sample	
	Standard	Inexperienced
Annual household income		
< 20k	8.6	12.7
20-39k	27.2	22.0
40-59k	29.1	17.3
60-79k	17.9	15.3
80-99k	6.6	12.0
100+k	10.6	20.7
Marital status		
Married	33.3	44.0
Separated	2.0	4.7
Widowed	0.7	-
Divorced	7.3	7.3
Never married	56.7	44.0
Children		
Yes	37.1	44.0
Race		
White	83.3	72.7
Black	8.7	12.7
Asian	3.3	6.0
Biracial	3.3	3.3
Other	1.3	5.3
Highest Degree		
No college degree	43.0	32.0
College degree	48.4	48.7
Post-college degree	8.6	19.3
Political party		
Republican	17.9	17.3
Democrat	51.7	44.0
Independent	29.8	26.7
Other	0.7	2.7
No preference	-	9.3
Religion		
Buddhist	2.6	-
Christian	34.4	50.7
Muslim	0.7	1.3
Jewish	4.0	-
Agnostic	23.8	14.7
Atheist	27.8	14.7
Other	5.3	10.0
Prefer not to say	1.3	8.7

*Note*: College degree = 2 or 4 year degree

Similar to Study 1, we setup and managed our studies using the TurkPrime platform. We limited participation to workers based in the US and employed TurkPrime’s tool to block submissions from workers using a VPN. All participants completed the same measures described in Study 1. Data collection for the standard sample finished in four hours and thirty minutes while data collection for the inexperienced sample finished in five hours.

## Results

### Data quality

#### Attention check questions

Table 10 shows the percentage of participants in each sample that passed all four attention check questions. Participants in the standard sample performed extremely well, with 98.6% passing three of the four questions. In the inexperienced sample, 92% of participants passed three of the four questions. For the question with the clearest scoring criteria, we found both groups passed the question at a high rate but participants in the standard sample (100%) were more likely to select the right answer than those in the inexperienced sample (92.7%).

**Table 10:** The percentage of participants who passed the attention check questions in Study 2.

Sample	Passed all ACQs	Number of ACQs failed			
		1	2	3	4
Standard	90.7	7.9	0.7	0.7	0.0
Inexperienced	78.8	13.2	5.3	2.6	0.0

*Note*: ACQ = attention check question

#### Completion time

We excluded one person from the timing analysis whose timing data said it took 139 minutes to complete the study (11 standard deviations above the mean). Afterward, the data indicated that people completed the study in 15.86 (*SD* = 10.98) minutes on average. However, the time to complete the study significantly differed between groups, *t*(299) = -7.27, *p* < .001,. People in the inexperienced sample took significantly longer to complete the study in minutes (*M* = 18.69, *SD* = 8.38) than those in the standard group (*M* = 12.21, *SD* = 7.03).

#### Exposure rates

As expected, people reported being more naïve in the inexperienced sample than the standard sample (see Table 11). Once again, differences in prior exposure were particularly striking for the CRT, Trolley Dilemma, and Asian Disease problem.

**Table 11:** The percentage of participants reporting prior exposure to the experimental measures in Study 2.

Sample	Experimental Task			
	Cognitive Reflection Task	Trolley Dilemma	Asian Disease	Mt. Everest
Standard	85.4	75.5	45.0	13.9
Inexperienced	20.5	9.9	2.0	3.3

#### Squared discrepancy scores

We subjected the data to the same analysis as in Study 1. As seen in Table 12 below, both groups were similarly consistent in their responses to the longest questionnaire in the study (Higher z-scores indicate more consistent responding). An independent samples *t*-test showed that the inexperienced group (*M* = 94.12, *SD* = 6.11) and the standard group (*M* = 93.67, *SD* = 9.73) were not significantly different, *t*(300) = -0.48, *p* = .64.

**Table 12:** Comparison of squared discrepancy scores across sample groups in Study 2.

Z-scores	Below 1		Between 1–2		Between 2–3		Between 3–4		Above 4	
Sample	*N*	*% of Sample*	*N*	*% of Sample*	*N*	*% of Sample*	*N*	*% of Sample*	*N*	*% of Sample*
Standard sample	7	4.8	9	6.0	19	12.6	48	31.8	68	45.0
Inexperienced	3	2.0	9	5.9	17	11.2	86	56.6	36	23.7

### Experimental results

#### Asian disease

The response frequencies for the Asian Disease experiment are presented in Table 13. As shown, responses to framing yielded similar results in all cases except for one: the Negative frame in the Standard sample. A logistic regression examining the condition by group interaction (Condition effect [% who chose program A in the Positive Frame—% who chose program A in the Negative Frame] standard = 32.3%, inexperienced = 36.4%) was not statistically significant when comparing the inexperienced sample to the standard sample (Logistic regression OR = -1.14, *p* = .80).

**Table 13:** The percentage of participants choosing each response option in the Asian Disease experiment in Study 2.

Sample	Positive Frame		Negative Frame	
	Response A	Response B	Response A	Response B
Standard	76.3*	23.7	44.0	56.0
Inexperienced	64.0*	36.0	27.6*	72.4

Note:

* Indicates a statistically significant χ^2^ at the *p* < .05 level, relative to a H_o_ value of 50%.

#### Mt. Everest

Similar to Study 1, a few participants entered extreme estimates for the height of Mt. Everest (e.g., 500,000 ft.), presumably the result of a typo. When looking at all the data, the anchoring manipulation worked in both groups: standard *t*(148) = 4.39, *p* < .001, *d* = 0.72, 95% CI [0.38, 1.05], inexperienced, *t*(148) = 5.69, *p* < .001, *d* = 0.93, 95% CI [0.59, 1.26]. Furthermore, a regression analysis revealed a large overall difference in people’s estimates based on anchoring condition, *b* = 39,068, *t* = 5.39, *p* < .001, 95% CI [24,800, 53,335], but no overall difference between the standard and inexperienced groups, *b* = 4,367, *t* = 0.60, *p* = .55, 95% CI [–9,946, 18,681], nor a significant interaction, *b* = -10,012, *t* = -0.98, *p* = .33, 95% CI [–30,188, 1,063]. This suggests the manipulation produced a similar effect in both the standard and inexperienced groups.

**Fig 7:**
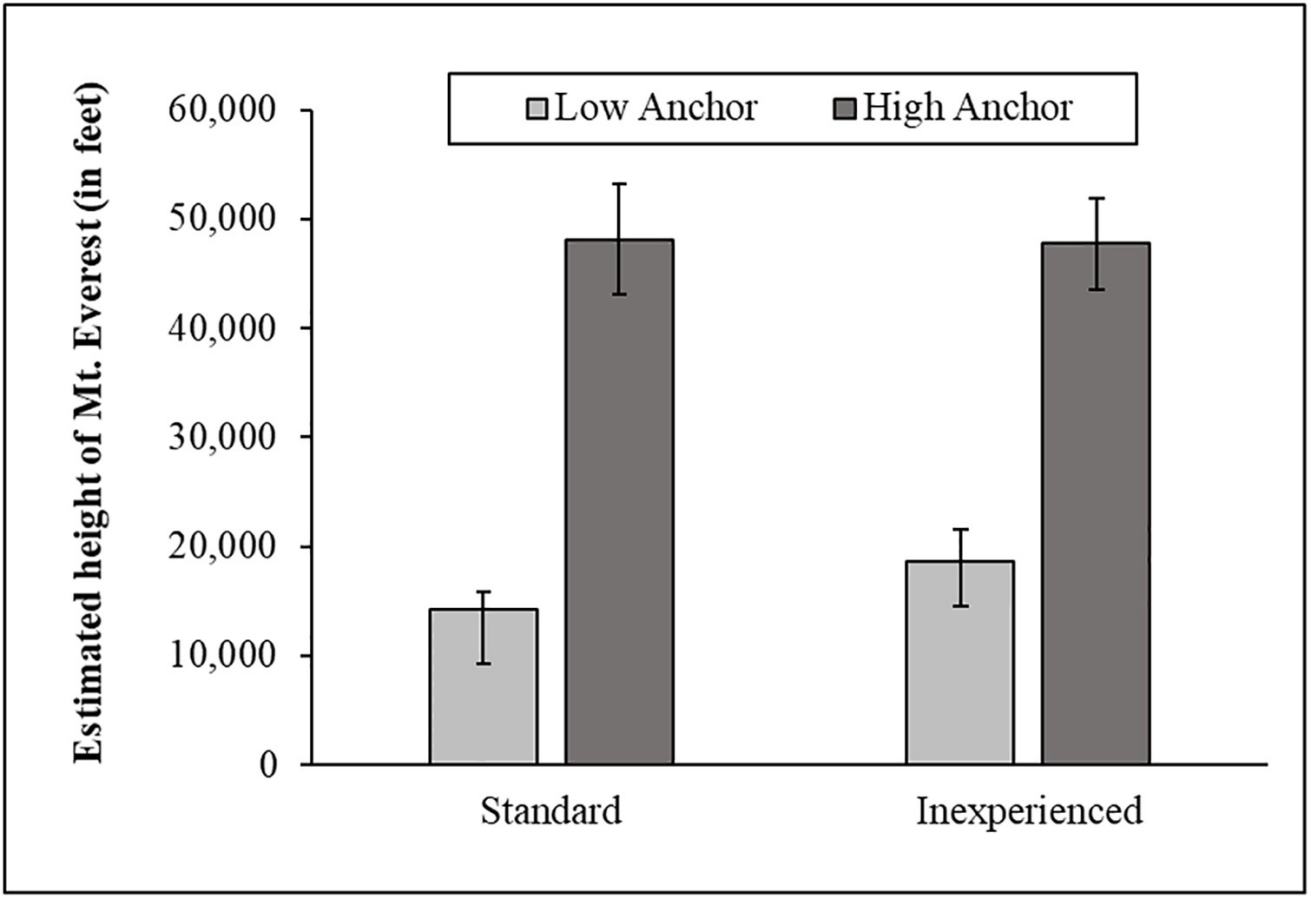
The anchoring manipulation across groups in Study 2.

Winsorizing estimates greater than five standard deviations from the mean yielded similar results. The anchoring manipulation worked in both groups: standard *t*(148) = 6.31, *p* < .001, *d* = 1.04, 95% CI [0.69, 1.37], inexperienced, *t*(148) = 5.69, *p* < .001, *d* = 0.93, 95% CI [0.59, 1.26] (see Fig 7). And, a regression analysis revealed a large overall difference in people’s estimates based on anchoring condition, *b* = 31,285, *t* = 7.88, *p* < .001, 95% CI [23,470, 39,100], but no overall difference between the standard and inexperienced groups, *b* = 4,681, *t* = 1.17, *p* = .24, 95% CI [–3,210, 12,572], nor a significant interaction, *b* = -3,814, *t* = -0.68, *p* = .50, 95% CI [–14,921, 7,291].

#### Trolley dilemma

The response frequencies for the Trolley dilemma experiment are presented in Table 14. The magnitude of the trolley dilemma effect as measured by a condition by group interaction (Condition effect [Yes response to the Classic dilemma—Yes response to the Footbridge dilemma] standard = 48.3%, inexperienced = 61.4%) was not significant when comparing the inexperienced to the standard sample (Logistic regression OR = 0.46, *p* = .16). The experimental effect replicated in both samples, and as indicated in Table 15, the effect size was somewhat larger in the inexperienced sample. Table 15 displays the effect sizes for both groups in all three experiments.

**Table 14:** The percentage of participants choosing each response option in the trolley dilemma in Study 2.

Sample	Version of Dilemma			
	Classic		Footbridge	
	No	Yes	No	Yes
Standard	26.0	74.0*	74.3*	25.7
Inexperienced	16.7	83.3*	78.1*	21.9

Note:

* Indicates a statistically significant χ^2^ at the *p* < .05 level, relative to a H_o_ value of 50%.

**Table 15:** Effect sizes of sample groups across experimental tasks in Study 2.

Sample	Experimental Task		
	Asian Disease (odds ratio)	Mt. Everest (Cohen’s d)	Trolley Dilemma (odds ratio)
Standard	4.09	1.04	8.26
Inexperienced	4.66	.93	17.21

#### BFI scores

Alpha reliability coefficients are presented in Table 16. Participants in the inexperienced group were similar to the standard group in the neuroticism and extraversion dimensions, and somewhat lower in the openness, conscientiousness, and agreeableness dimensions. The reliabilities for the inexperienced group were virtually identical to a previous internet sample [1].

**Table 16:** Alpha coefficients for dimensions of the BFI in Study 2.

Dimension	Sample		
	Standard	Inexperienced	Buhrmester et al., 2011
Openness	.86	.78	.79
Conscientiousness	.90	.81	.77
Extraversion	.92	.85	.87
Agreeableness	.87	.79	.77
Neuroticism	.94	.85	.85

#### CRT scores

CRT scores differed significantly between the groups: standard (*M* = 2.03, *SD* = 1.19), inexperienced (*M* = 0.83, *SD* = 1.08), *b* = -1.21, *t* = -9.23, *p* < .001, 95% CI [-1.46, -0.95]. As in Study 1, we interpret this finding as further evidence that people who have previously seen the CRT answer more questions correctly than those who have not. Indeed, previous exposure was again positively correlated with performance (*r* = .33, *p* < .001).

### Discussion

Similar to Study 1, the results of Study 2 demonstrated that inexperienced workers provide high quality data across multiple indicators including attention checks, internal reliability of a personality questionnaire and effect sizes of experimental manipulations. Across measures, the results of Study 2 suggest that inexperienced workers are a source of quality data. Most importantly, our results indicate that researchers can sample this group by changing standard sampling practices.

## General discussion

Amazon Mechanical Turk is no longer a novel source of research participants within the social sciences. One benefit of researchers’ sustained interest in the platform over time is that a number of studies have accumulated suggesting ways for researchers to avoid common pitfalls and plan around common concerns when using MTurk [12]. In this paper, we add to this literature by proposing a method of sampling from MTurk that alleviates one of the most persistent concerns researchers have voiced about the platform: the problem of participant non-naivete. Our data show that approximately 70% of workers are relatively inexperienced, that new workers join the platform at a rapid pace, and that researchers can collect quality data when targeting inexperienced workers. We focus our discussion on the trade-offs of different sampling practices on MTurk, recommendations for researchers who want to recruit a more diverse sample of workers, and directions for future research.

### Inexperienced workers as a viable alternative

The belief that standard worker qualifications are necessary to collect quality data on MTurk is deeply held. Part of the reason for this is that data quality is critical when conducting research online. However, we suspect that researchers adhere to worker qualifications because it is the answer they have come to know as the standard response to data quality concerns on MTurk [18]. Each time questions about data quality threaten researchers’ confidence in the platform, the proposed remedy is to selectively sample workers with a high approval rating. For example, after a threat to data quality on MTurk in the summer of 2018, a group of researchers writing in the *Washington Post* stated that to ensure data quality on MTurk researchers should, “follow the standard best practices when conducting MTurk research. That includes setting the “HIT [an MTurk term for ‘task’] Approval Rate (%)” above 95 percent and the “Number of HITs Approved” to at least 100, which substantially improves data quality" [41]. Similar advice was common on message boards and social media.

As our data show, however, using standard worker qualifications to ensure data quality comes with the cost of sampling very experienced workers (Fig 3). Furthermore, this is a trade-off that researchers do not have to make. In both Studies 1 and 2, we found that workers with less than 50 HITs completed provided quality data that was similar to a standard sample in most respects. Inexperienced workers had acceptable reliability scores on the BFI, high scores in the squared discrepancy procedure, large effect sizes on each experimental task, and less previous exposure to all the measures in our study than the standard sample.

Across both studies, the inexperienced group had fewer participants who responded randomly than the standard sample. Additionally, participants in the standard sample completed the study much faster than participants in the inexperienced sample. These results are consistent with prior research showing that experienced participants complete studies faster than inexperienced ones [14]. These results also suggest that there is little reason to avoid inexperienced workers for fear that they provide bad data quality. Of course, despite our effort to examine data quality in many different ways, it is possible that inexperienced workers may differ from experienced workers in data quality in ways that are yet unknown. For example, we only examined whether three well-known experimental effects replicate. Although these effects did replicate and require attention to the words of each manipulation, other manipulations may yield different results.

Additionally, on some measures of attentiveness in Study 2 the standard sample appeared to perform slightly better than the inexperienced sample. The standard sample passed attention checks at a slightly higher rate than the inexperienced sample and produced higher reliability coefficients on the BFI. However, in extreme cases, using standard worker qualifications may mean that researchers are continually sampling from less than 5,000 superworkers who have completed a disproportionately high number of social science studies and who past research has shown perform well on attention checks and measures of reliability because they *learn* from prior experience [18]. Our research demonstrates that inexperienced workers are a viable alternative.

### The trade-offs of different sampling practices on MTurk

Our recommendation is not that highly active and experienced workers should never be sampled. Instead, we believe researchers should sample these workers when the purpose of a study is suited to experienced participants. When might this be? Although there is currently little to no research on which to base this decision, it seems likely that experienced workers are more willing to engage in complicated or tedious tasks than inexperienced workers and are much less likely to attrit from longitudinal studies [42–43]. In addition, because experienced workers are consistently available on MTurk they are likely well-suited to studies that require extensive participant engagement. Studies that ask workers to complete tasks that last several hours, engage in experience sampling over several days, or collaborate with other workers to solve novel problems may all be better suited to superworkers than to new workers [43].

For most other studies researchers can sample inexperienced workers by using the HIT completion requirements to exclude the most active participants. Based on the results presented here, inexperienced workers seem well suited for short surveys and simple experimental manipulations similar to what was included in our studies. Inexperienced workers are likely good participants for a broad swath of social science research, but there is no current data to suggest they will provide quality responses on long open-ended questions or engage in tasks that last a long time (e.g., an hour or several hours). Future studies should investigate these questions by comparing data quality of workers with various levels of experience on many different types of tasks that have not been examined in this study. Figuring out how much engagement researchers can get from inexperienced workers is an opportunity for future research.

### A potential objection to sampling inexperienced workers: The speed of data collection

Although researchers may want to sample inexperienced workers, a question that arises from our studies is whether inexperienced workers are active enough to support a change in most researchers’ sampling practices toward favoring inexperienced workers over experienced ones. A partial answer to this question comes from the time it took to collect data in the present studies. In both studies, data collection for the inexperienced sample completed in the same amount of time as the standard sample.

**Fig 8:**
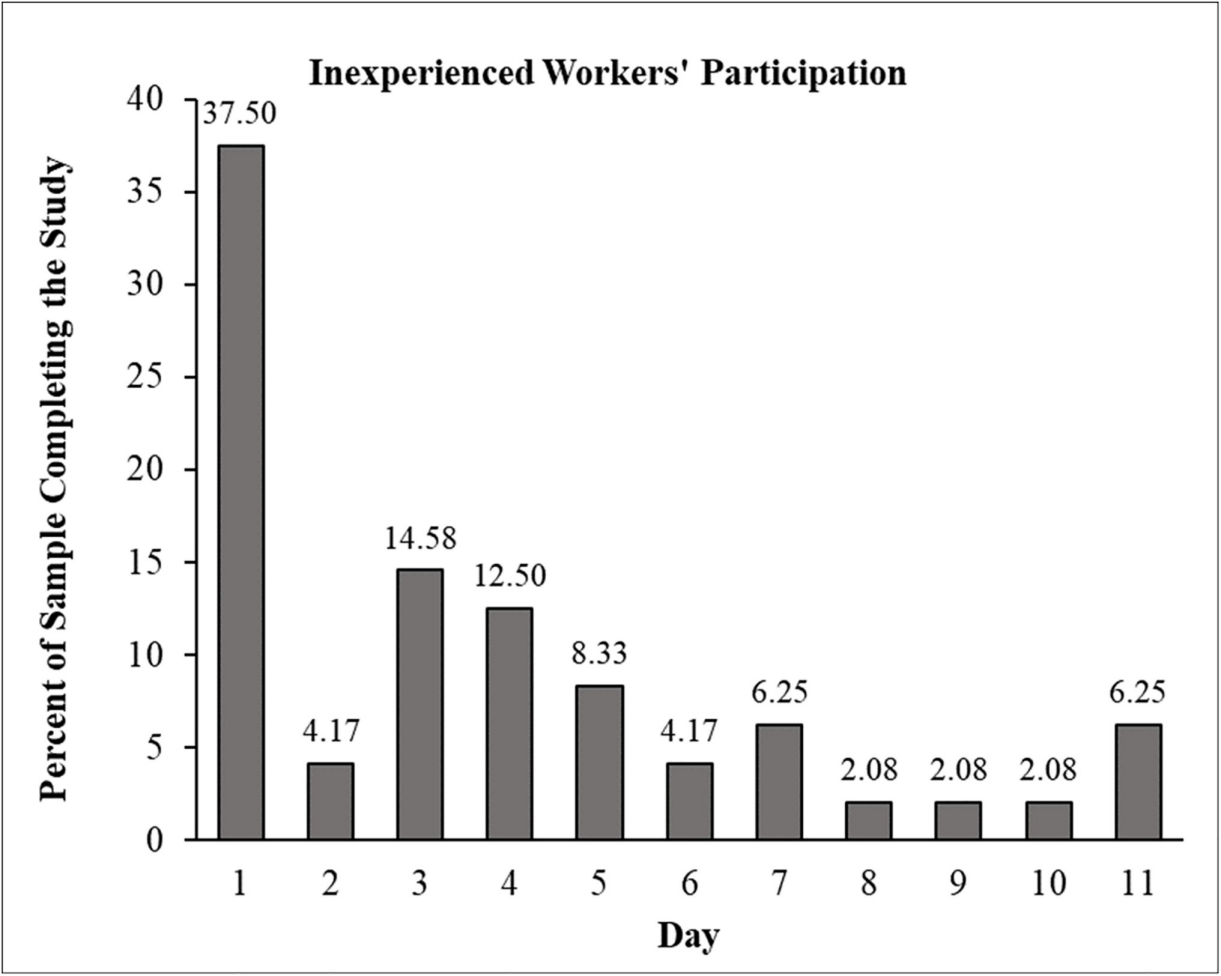
The percent of inexperienced workers participating in Study 3 by day. In total, 48 participants completed the study.

To further assess whether inexperienced workers are active enough to support speedy data collection on a larger scale, we conducted a short study in which we randomly sampled inexperienced workers. Our goal was to estimate what percentage of inexperienced workers can be expected to take a study within a given interval. To do so, we queried the TurkPrime database in October 2018 and compiled a list of all workers who had completed at least one HIT but not more than 100 HITs within the last 30 days. The list included 17,198 workers. Next, we randomly selected 100 workers from the list and allowed them, and only them, to take our study. During the days our study was open, 49 workers started the study and 48 completed it. Looking at the data over time, we found almost 40% of workers took the HIT on Day 1, and 78% took the study within 5 days (Fig 8). Altogether, we believe this data provides a reason to be optimistic about the speed of data collection with inexperienced workers. Even among the least active workers, 50% can be expected to participate in a study if given the opportunity. Considering that such workers constitute close to 70% of the participant pool, these results show that MTurk is full of untapped potential.

### General recommendations for how to sample workers on Mechanical Turk

A key question raised by our findings is, how should researchers sample Mechanical Turk workers? The most immediate implications of our results are that researchers should find ways to allow less experienced workers into their studies and restrict the percentage of highly experienced workers in their sample. We suggest two ways to do this.

First, researchers could use quotas to control the number of experienced and inexperienced workers who can participate in a study. As Fig 3 shows, the most active 11% of workers complete over 60% of HITs and the most active 28% complete close to 90% of HITs. Although there are several ways researchers could slice up their sample, one way would be to open 30% of spots to workers with more than 1,000 HITs and reserve 70% of spaces for the nearly 70% of workers with less than 1,000 previous HITs completed. Stratifying a sample along these lines—or several other combinations of worker experience—would have the benefit of maintaining the speed of data collection while balancing the participation of highly experienced and active workers against the participation of inexperienced and naive workers.

A second way to recruit inexperienced workers is to set a threshold of experience and to target workers below that threshold. For example, researchers could decide to sample only workers with fewer than 5,000 or 100 or any other number of previously completed HITs. The advantage of this strategy is that it easily solves the issue of participant non-naivete. The downsides, however, are that this strategy may slow data collection, particularly for large studies, and raises questions about the wisdom of excluding all workers above a certain threshold. As a guideline, we believe that for many studies run on MTurk, excluding the most active 11% of workers who have taken 5,000 HITs or more would still allow data to come in quickly, while removing the extremely active and non-naive workers from a sample. However, as we have mentioned, experienced workers are likely well-suited for a number of studies within the social and behavioral sciences. Thus, researchers should consider on a study-by-study basis how and why to limit the participation of experienced workers.

Regardless of how researchers choose to limit the participation of experienced workers, we recommend special consideration to the participation of the most inexperienced workers. This is because the most inexperienced workers on MTurk—those with fewer than 100 previously completed HITs—are likely less active than workers with more experience. Therefore, wherever researchers decide to set the bar on worker experience, say 5,000 HITs, the participants who complete the study are likely to congregate toward the upper bound of this threshold. As a result, we recommend that researchers explicitly reserve some portion of their study for workers with fewer than 100 HITs completed when the goal is to sample naïve workers.

Related to the issue of how researchers should use the qualification of previous HITs completed is the question of how to use the approval rating qualification. Although our research suggests there are few workers with a low approval rating on MTurk, we recommend that researchers set an approval rating of at least 95% for one simple reason. Requiring that workers have a high approval rating is an incentive for most people to provide quality responses. When requesters reject poor or fraudulent work as they should, the approval rating serves as a reputation mechanism that ensures quality data.

## Conclusion

Over the last ten years, Mechanical Turk has become a common source of research participants. At the same time, researchers have worried about workers becoming increasingly non-naive. In this article, we demonstrated that contrary to the thinking that suggests MTurk is a tapped-out resource, in reality, the opposite is true: MTurk is a vast resource with untapped potential researchers can capitalize on by changing the way they use the platform. Specifically, by changing the way worker qualifications are currently used, researchers can exclude the most experienced workers and target inexperienced workers who provide quality data. Targeting less experienced workers will significantly increase the available pool of MTurk workers, mitigate the superworker problem, and help resolve the issues of non-naivete all while allowing researchers to benefit from the advantages that originally made MTurk an attractive source of research participants.
