# Correction: Tapped out or barely tapped? Recommendations for how to harness the vast and largely unused potential of the Mechanical Turk participant pool

**DOI:** 10.1371/journal.pone.0342218

**Published:** 2026-02-04

**Authors:** Jonathan Robinson, Cheskie Rosenzweig, Aaron J. Moss, Leib Litman

The OSF link in this article’s [1] Data Availability statement is incorrect. The correct link is: https://osf.io/9e476/overview.
